# ADS-MIR: A Machine Perception-Oriented Visible-Infrared Sensor Fusion Framework for Intelligent Transportation Perception Under Complex Illumination Conditions

**DOI:** 10.3390/s26123675

**Published:** 2026-06-09

**Authors:** Jun Yang, Jianguo Wu, Xiaolan Zhang, Zenglong Yang, Hongfei Shen, Botao Shen, Chang Zeng

**Affiliations:** 1 Big Data and Internet of Things Research Center, China University of Mining and Technology, Beijing 100083, China; yj@cumtb.edu.cn (J.Y.); zqt2310405044@student.cumtb.edu.cn (X.Z.); zqt2310405043@student.cumtb.edu.cn (Z.Y.); sqt2410405033@student.cumtb.edu.cn (B.S.); sqt2510405047@student.cumtb.edu.cn (C.Z.); 2 Research Institute of Highway, Ministry of Transport, Beijing 100088, China; hf.shen@rioh.cn

**Keywords:** multimodal sensor fusion, infrared and visible image fusion, intelligent transportation systems, object detection, illumination-adaptive sensing, edge information transfer

## Abstract

Multimodal sensor fusion in intelligent transportation systems faces severe challenges in maintaining reliable visual information acquisition under complex illumination conditions. Extreme low-light and intense glare significantly degrade visible-light sensor imaging quality, making it difficult for single-modal vision systems to maintain reliable target perception. Meanwhile, although infrared sensors provide a relatively stable saliency complement for target regions, modal discrepancies and spatial misalignment between heterogeneous visible and infrared sensors often degrade fusion performance, limiting the practical benefits of multimodal sensing for machine perception. To address these issues, this study proposes Aligned, Dual-Gated, and Saliency-Guided MIRNet (ADS-MIR), a machine perception-oriented visible-infrared sensor fusion framework that enhances the discriminability and structural representation of target regions for roadside perception sensors operating under complex conditions. Specifically, the framework employs a domain alignment layer to mitigate feature distribution discrepancies and spatial misalignment between heterogeneous sensor modalities. An illumination-guided adaptive gating mechanism dynamically modulates bimodal sensor feature contributions, while a saliency-guided frequency decoupling reinforcement strategy reinforces target-related high-frequency edge details. Experimental results on the LLVIP and M3FD datasets demonstrate that ADS-MIR improves the edge information transfer factor ($Q^{AB/F}$) by 49.6% to 111.6% compared with existing methods, highlighting its distinct advantage in preserving target contours and restoring edge information. Furthermore, the enhanced results provide more discriminative input features for downstream object detection, exhibiting more stable perception capabilities under complex illumination and challenging sensing scenarios.

## 1. Introduction

Robust multimodal sensor fusion under complex environmental conditions is a critical requirement for the practical deployment of intelligent transportation systems (ITSs), where heterogeneous roadside sensing units must maintain reliable perception across diverse and unpredictable illumination scenarios. Particularly in extreme lighting scenarios—such as nighttime low light, tunnel transitions, intense glare from oncoming vehicles, rain- or fog-induced scattering, reflective road surfaces, and local overexposure caused by strong headlights—visible-light images often suffer from a decreased signal-to-noise ratio (SNR), restricted dynamic range, and degraded target details [1]. In contrast, infrared (IR) imaging provides relatively stable target saliency information even in weak or zero-illumination conditions, serving as an important complement for visual perception. However, IR images typically lack rich texture and color representations, and heterogeneous visible and infrared sensors may also suffer from residual spatial offsets due to different viewpoints, imaging mechanisms, and calibration errors. Consequently, effectively fusing visible and infrared images to construct visual representations more conducive to machine perception has become an important research direction for robust ITS perception [2,3].

Nevertheless, infrared saliency is not always equivalent to semantic targetness. In roadside environments, heated asphalt, building facades, vehicle exhaust regions, reflective surfaces, and other non-target thermal responses may generate strong infrared activations. If such responses are directly injected into visible-light features without reliability control, then they may introduce artifacts or mislead downstream perception models. Therefore, visible-infrared fusion for ITSs should not only integrate complementary information but also estimate modality reliability, suppress unreliable high-frequency noise, and reinforce target-related structures in a controlled manner.

Although recent representation learning paradigms have driven significant progress in image enhancement and multimodal fusion, existing methods still face two key limitations when serving downstream machine perception tasks. First, there is a mismatch between human-oriented visual enhancement and machine-oriented perception. Many enhancement algorithms favor visually smooth and natural-looking outputs, but this smoothing tendency may weaken the high-frequency structures and sharp boundaries that detectors rely on [4,5]. Thus, human perception-oriented metrics such as visual information fidelity (VIF) and structure-oriented metrics such as the edge information transfer factor ($Q^{AB/F}$) may reflect different optimization preferences. Second, the heterogeneity of multimodal integration is still insufficiently addressed. When visible and infrared features exhibit discrepancies in statistical distributions, response mechanisms, and spatial alignment, direct concatenation or fixed-weight superposition can produce unstable fused representations. Under drastic illumination fluctuations, unreliable visible-light responses or false thermal activations may further contaminate the fused features. Therefore, cross-modal alignment, illumination-adaptive fusion, and task-consistent structural enhancement remain challenging for visible-infrared ITS perception.

To address these bottlenecks, this paper proposes Aligned, Dual-Gated, and Saliency-Guided MIRNet (ADS-MIR), a machine perception-oriented visible-infrared multimodal image enhancement framework. Recognizing the physical limitations of single-modal enhancement in extreme low-light and severe glare scenarios, we build upon MIRNet-v2 [6] to construct a dual-stream multimodal architecture. Although MIRNet-v2 provides strong high-resolution representation capability for image restoration, the original architecture is designed mainly for single-modal restoration and does not explicitly address cross-modal distribution discrepancy, residual RGB–IR misalignment, illumination-dependent modality reliability, or infrared-guided target boundary reinforcement. Unlike traditional enhancement methods driven primarily by human visual perception, ADS-MIR prioritizes the discriminability and structural quality of target regions under complex sensing conditions. The framework follows an alignment–gating–fusion–reinforcement logic to mitigate modality discrepancy, dynamic illumination interference, and objective mismatch, while remaining detector-agnostic and not requiring joint detector training. The main contributions of this paper are summarized as follows:

1. A machine perception-oriented multimodal enhancement framework: We propose ADS-MIR, a visible-infrared enhancement framework built upon MIRNet-v2 and extended from single-modal restoration to dual-stream multimodal perception. It improves target-related structural representations under low light, glare, and residual sensor mismatching through an alignment–gating–fusion–reinforcement pipeline.
2. A cross-modal reliability modeling and fusion strategy: We introduce a domain alignment layer (DAL) to reduce first-order statistical discrepancies between heterogeneous RGB and IR features. An Illumination-Guided Adaptive Gating (IGAG) module estimates illumination reliability and softly modulates infrared contribution, while frequency-aware fusion (FAF) regulates low-frequency compensation and high-frequency detail injection.
3. A saliency-guided structural reinforcement mechanism: We design a Saliency-Guided Frequency Decoupling Reinforcement (SGFD-R) module to reinforce target-related high-frequency boundaries using infrared saliency priors. This module prioritizes structural discriminability over purely smoothing-oriented visual naturalness while avoiding global amplification of all infrared responses.
4. Comprehensive mechanism-level and task-level validation: Experiments on LLVIP and M3FD show that ADS-MIR achieves a relative improvement from 49.6% to 111.6% in $Q^{AB/F}$ over representative fusion methods. We further provide inference-time IGAG gate analysis, image-level $Q^{AB/F}$ confidence intervals, class-wise downstream detection analysis, and computational cost discussion under a fixed 256×256 RGB–IR input setting.

## 2. Related Work

### 2.1. Low-Light Image Enhancement and Restoration Backbones

To restore visibility under degraded environmental conditions, existing low-light image enhancement methods mainly rely on Retinex-based physical models or data-driven learning networks. Traditional physics-based methods [7] enhance contrast by decomposing illumination and reflectance components, but they may amplify background noise and introduce color distortion in extremely dark scenes. Deep Retinex architectures [8,9], self-supervised enhancement methods [10,11], and self-calibrated lighting learning [12] have improved low-light restoration by learning adaptive enhancement mappings. In addition, task-oriented enhancement pipelines [4,13] have attempted to improve downstream detector robustness under adverse illumination. Recently, Mahdizadeh et al. [14] proposed a Mamba-based progressive-recovery framework for multimodal low-light image enhancement, showing the potential of state-space modeling for progressive cross-modal restoration. Mu et al. [15] introduced an illumination-adaptation strategy for SAM to improve segmentation accuracy in low-light scenes, further indicating that low-light enhancement and illumination-aware adaptation are important for downstream perception tasks.

Recent restoration backbones such as MIRNet [16] and MIRNet-v2 [6] preserve high-resolution spatial representations while aggregating multi-scale contextual information, making them effective foundations for image restoration. However, MIRNet-v2 is primarily designed for single-modal restoration and does not explicitly address cross-modal distribution discrepancy, residual RGB–IR spatial mismatching, illumination-dependent modality reliability, or infrared-guided target boundary reinforcement. Therefore, applying MIRNet-v2 to visible-infrared roadside perception requires extending it from a single-modal restoration backbone into a dual-stream multimodal fusion framework.

Despite these advances, single-modal enhancement remains limited under near-zero illumination or intense glare, where visible-light sensors may lose or saturate critical texture information. This limitation motivates the integration of infrared priors to improve perception robustness under complex illumination.

### 2.2. Visible-Infrared Image Fusion Under Complex Illumination

Visible-infrared image fusion provides a complementary strategy for overcoming visible-sensor failures, but conventional methods often struggle to balance visual aesthetics with machine perception requirements. Traditional multiscale transforms [17,18] aggregate information using predefined rules, which are difficult to adapt to drastic illumination changes and residual cross-modal misalignment. Such rigid fusion may produce ghosting artifacts or blur target boundaries, thereby affecting downstream perception.

Recent deep fusion methods, including GAN-based models, transformer-based dual-branch architectures [19], and state-space models [20,21], have improved global modeling and fusion quality. Illumination-aware networks [22] and adaptive energy scoring mechanisms [23,24] further attempt to dynamically weight visible and infrared features. Nevertheless, many existing methods still rely on global feature stacking or insufficiently constrained infrared injection, which may propagate thermal noise into high-frequency textures.

A practical challenge in roadside scenes is that infrared saliency is not always semantically reliable. Heated asphalt, building facades, vehicle exhaust regions, and reflective surfaces may generate strong non-target thermal responses. If these false thermal responses are directly injected into fused features, they may reinforce misleading structures or introduce artifacts. Therefore, robust visible-infrared fusion should estimate modality reliability and constrain infrared high-frequency reinforcement to target-related regions.

### 2.3. Machine Perception-Oriented Fusion and Downstream Perception

Most enhancement and fusion methods are still evaluated mainly from the perspective of human visual perception, including brightness, contrast, naturalness, and visual fidelity. However, downstream perception models rely more directly on target contours, local gradients, boundary sharpness, and discriminative structural cues. Therefore, human perception-oriented metrics such as visual information fidelity (VIF) and structure-oriented metrics such as the edge information transfer factor ($Q^{AB/F}$) may exhibit different trends under machine perception-oriented enhancement.

To bridge low-level fusion and high-level perception, recent studies have introduced semantic-aware losses or embedded fusion modules into downstream vision tasks [25]. These task-driven strategies can improve detector-specific performance, but they often require joint training with a particular detector or segmentation network, reducing flexibility across perception backends. In contrast, detector-agnostic fusion frameworks can be used as modular preprocessing units, but they must rely on general structural cues such as edge transfer, saliency preservation, and boundary reinforcement rather than detector gradients.

### 2.4. Computational Complexity and Roadside Deployment

Computational efficiency is critical for roadside sensing units (RSUs), which often operate continuously under limited hardware resources. Lightweight fusion networks can achieve fast inference but may lack sufficient capacity to handle cross-modal discrepancy, drastic illumination variation, residual mismatching, and target-aware structural reinforcement simultaneously. Conversely, more complex architectures may improve robustness and structural preservation, but their deployment cost must be explicitly analyzed. Therefore, practical visible-infrared perception in an ITS requires a trade-off between structural discriminability and computational efficiency. In this work, we report the trainable parameters, MACs and FLOPs, and inference speed under a fixed 256×256 RGB–IR input setting and discuss lightweight optimization directions for future RSU deployment.

## 3. Proposed Methods

Motivated by the limitations of the “open-loop” fusion paradigms discussed in Section 2, merely pursuing visual fidelity may compromise the critical edge gradients required by backend detectors. To bridge the gap between low-level physical reconstruction and high-level machine perception, we propose the Aligned, Dual-Gated, and Saliency-Guided MIRNet (ADS-MIR) framework. Unlike conventional methods, which indiscriminately stack multimodal features, ADS-MIR is explicitly designed as a machine perception-oriented architecture. It systematically decomposes the perception challenge in complex environments into four targeted mechanisms: mitigating sensitivity to spatial misalignment via a domain alignment layer (DAL), adapting to extreme lighting via Illumination-Guided Adaptive Gating (IGAG), separating spatial sub-bands via frequency-aware fusion (FAF), and preserving target-related local structures via Saliency-Guided Frequency Decoupling Reinforcement (SGFD-R). By integrating these modules through a multi-level progressive strategy, the framework helps the enhancement process provide more discriminative structural cues for downstream perception.

### 3.1. Framework Overview and Problem Description

**Figure 1:**
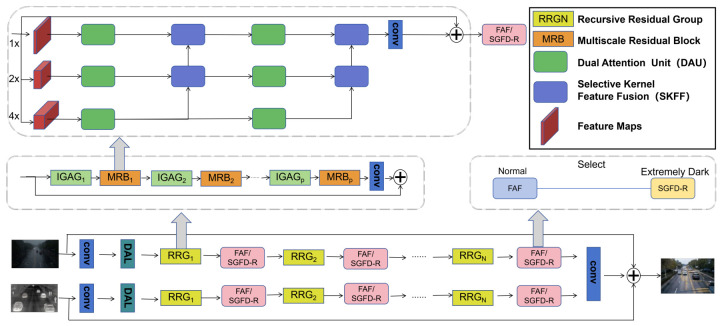
Overall architecture of the proposed ADS-MIR framework. Built upon MIRNet-v2, ADS-MIR adopts a dual-stream visible-infrared input and a multi-level progressive fusion strategy. The fusion chain follows an alignment–gating–fusion–reinforcement logic, where DAL performs feature alignment, IGAG estimates illumination-dependent modality reliability, FAF regulates low- and high-frequency responses, and SGFD-R reinforces target-related structural details.

ADS-MIR is designed as a detector-agnostic visible-infrared enhancement framework for roadside perception under complex illumination. As illustrated in Figure 1, the framework takes a visible-light image $F_{RGB}\in R^{H\times W\times 3}$ and an infrared image $F_{IR}\in R^{H\times W\times 1}$ as paired multimodal inputs and generates an enhanced image $\hat{I}$ that emphasizes target-related structural cues for downstream perception. Built upon MIRNet-v2 [6], ADS-MIR adopts a dual-stream architecture to extract visible and infrared features in parallel, followed by progressive cross-modal calibration.

The overall design follows an “alignment–gating–fusion–reinforcement” logic. First, the domain alignment layer (DAL) reduces statistical discrepancies between heterogeneous RGB and IR features before cross-modal interaction. Second, the Illumination-Guided Adaptive Gating (IGAG) module estimates visible-light reliability and generates a soft infrared gate under varying illumination. Third, the Frequency-Aware Fusion (FAF) module regulates low-frequency illumination compensation and high-frequency detail injection through lightweight spatial sub-band approximation. Finally, the Saliency-Guided Frequency Decoupling Reinforcement (SGFD-R) module uses infrared saliency as a soft spatial prior to reinforce target-related local structures while avoiding global amplification of all infrared responses.

This fusion chain is embedded within the recursive residual groups (RRGs) of the MIRNet-v2 backbone to form a multi-level progressive fusion strategy. Compared with single-pass terminal fusion, the progressive strategy enables multimodal calibration at different feature depths, allowing shallow layers to stabilize low-level contours and deeper layers to accumulate illumination-aware and saliency-guided structural priors. ADS-MIR therefore serves as a perception-oriented preprocessing module rather than a detector-specific end-to-end model.

Under this structural paradigm, the enhancement of low-light road images is formalized as a pixel-level mapping problem. Given the paired inputs $F_{RGB}$ and $F_{IR}$, the objective is to generate an enhanced image $\hat{I}$ that minimizes the distance to a clear reference image $I^{*}$ in the pixel space. Following the robust restoration paradigm established in [6], we employ the Charbonnier loss function as the optimization objective

(1) $$L\left(\hat{I},I^{*}\right)=\sqrt{{∥\hat{I}-I^{*}∥}^{2}+\epsilon^{2}}$$

where $\epsilon =10^{-3}$ is a smoothing constant. Compared with the standard $L_{2}$ loss, this function is more robust to outlier pixels and helps prevent stochastic noise from destabilizing gradient updates.

It should be noted that the machine perception-oriented attribute of ADS-MIR is mainly reflected in its structural design rather than in a detector-specific training objective. All internal components are jointly optimized under the global image restoration objective. This detector-agnostic design improves modularity and deployment flexibility, while task-driven joint optimization with downstream detectors is left as a future extension.

### 3.2. Domain Alignment Layer via Instance Normalization

Physical displacement between visible and infrared sensors in roadside sensing systems can introduce residual spatial offsets even after preliminary geometric registration. In addition, visible and infrared images are generated by different imaging mechanisms, which leads to inconsistent feature statistics, response distributions, and intensity scales. If such heterogeneous features are directly aggregated, then cross-modal discrepancy may amplify unstable responses and produce “ghosting” artifacts around object boundaries.

The proposed domain alignment layer (DAL) is designed as a lightweight feature-level statistical alignment step before cross-modal fusion. It does not aim to perform explicit pixel-level geometric registration; instead, it reduces modality-specific first-order statistical discrepancies, such as the mean shift, contrast scale, and response magnitude, so that the subsequent gating and fusion modules can operate on a more stable feature basis. This design is particularly suitable for visible-infrared roadside sensing, where residual spatial mismatch and heterogeneous modality statistics often coexist.

Instance normalization (IN) is adopted in the DAL because it performs sample-wise and channel-wise normalization over the spatial domain. This property is consistent with the goal of instance-level modality calibration: for each paired RGB–IR sample, the DAL weakens the absolute statistical differences while preserving learnable affine flexibility through trainable parameters. Therefore, the DAL should be understood not as a new normalization operator but as a targeted statistical calibration component inserted into the progressive RGB–IR fusion pipeline.

For an input feature map $F\in R^{C\times H\times W}$ of each modality, the channel-wise mean μ(F) and standard deviation σ(F) are computed first:

(2) $$\mu \left(F\right)=\frac{1}{HW}\sum_{h=1}^{H}\sum_{w=1}^{W}F_{c,h,w},$$

(3) $$\sigma \left(F\right)=\sqrt{\frac{1}{HW}\sum_{h=1}^{H}\sum_{w=1}^{W}{\left(F_{c,h,w}-\mu \left(F\right)\right)}^{2}+\epsilon }.$$

Subsequently, regularized calibration across domains is achieved through a standardized mapping function:

(4) $$F_{aligned}=\gamma \cdot \frac{F-\mu (F)}{\sigma (F)+\epsilon }+\beta ,$$

where $\epsilon =10^{-5}$ is a stabilization constant, while γ and β are learnable affine parameters optimized during training.

By introducing this statistical alignment step before IGAG and FAF, the DAL provides a more comparable feature foundation for illumination-aware gating and frequency-aware fusion. Its effectiveness is empirically supported by the ablation study in Section 4.5, where adding a DAL to the dual-stream baseline substantially improves the edge information transfer metric $Q^{AB/F}$ on M3FD. The qualitative visualization also shows that the DAL reduces cross-modal ghosting and stabilizes object contours before subsequent structural reinforcement.

**Figure 2:**
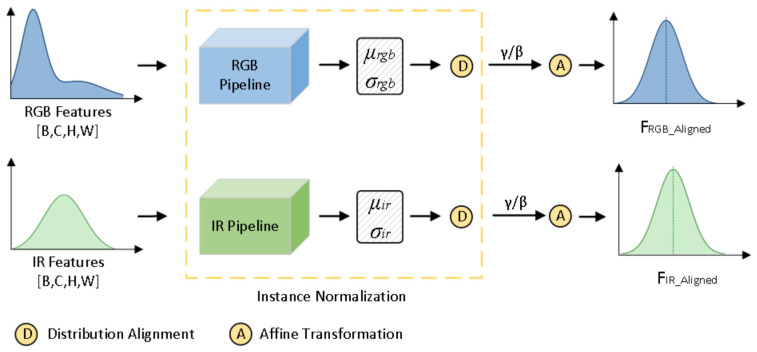
Detailed architecture of the domain alignment layer (DAL). The DAL performs sample-wise first-order statistical alignment on heterogeneous RGB–IR features before cross-modal aggregation. Rather than correcting pixel-level displacement, it reduces modality-specific feature distribution discrepancy and alleviates the sensitivity of subsequent fusion modules to residual spatial mismatching.

The detailed architecture of the DAL is illustrated in Figure 2.

### 3.3. Illumination-Guided Adaptive Gating

**Figure 3:**
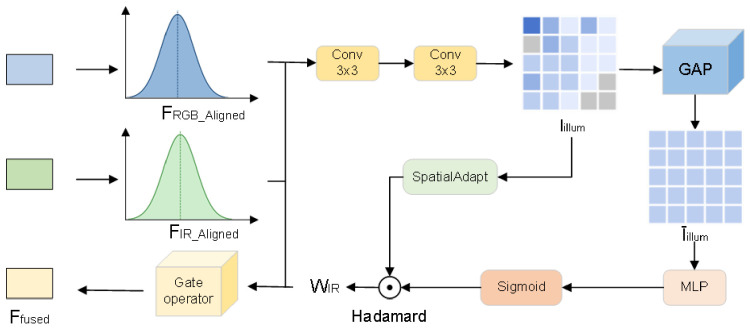
Detailed architecture of the IGAG module. IGAG estimates an illumination map $I_{illum}$ from aligned visible-light features and generates a soft infrared gate $w_{IR}$ through a low-illumination prior and a spatially adaptive branch. The gate modulates RGB–IR feature contributions in an illumination-aware and spatially adaptive manner, rather than using a fixed fusion ratio.

Conventional multimodal fusion methods frequently employ static fusion rules, which cannot adaptively modulate the contributions of heterogeneous modalities under fluctuating illumination. The proposed Illumination-Guided Adaptive Gating (IGAG) module implements an illumination-sensing and soft modulation pipeline, as illustrated in Figure 3. Its purpose is to avoid rigid RGB–IR fusion when the reliability of visible-light observations changes significantly. Instead of assigning a fixed fusion ratio to the two modalities, IGAG estimates an illumination prior from the aligned visible-light features and uses it to adaptively regulate the infrared contribution.

The module estimates a pixel-level illumination map $I_{illum}\in {\left[0,1\right]}^{B\times 1\times H\times W}$ from the aligned visible-light feature $F_{RGB\_Aligned}$ via consecutive 3×3 convolutional layers:

(5) $$I_{illum}=\sigma \left(\mathrm{Conv}\left(\mathrm{Conv}\left(F_{RGB\_Aligned}\right)\right)\right),$$

where σ denotes the sigmoid activation. Subsequently, a global average pooling (GAP) layer compresses $I_{illum}$ into a scalar illumination index ${\bar{I}}_{illum}\in {\left[0,1\right]}^{B\times 1\times 1\times 1}$:

(6) $${\bar{I}}_{illum}=\mathrm{AdaptiveAvgPool}2d\left(I_{illum}\right).$$

To account for the decay of visible-light effectiveness in dark conditions, we introduce a low-illumination prior into the infrared weight generation process:

(7) $$w_{IR}=\sigma \left(\mathrm{MLP}\left({\left(1-{\bar{I}}_{illum}\right)}^{2}\right)\right)⊙\mathrm{SpatialAdapt}\left(I_{illum}\right),$$

where $w_{IR}$ represents the infrared modal weight. The term ${\left(1-{\bar{I}}_{illum}\right)}^{2}$ becomes larger when the estimated visible-light illumination decreases, thereby providing a low-illumination prior that biases the learned gate toward assigning a larger contribution to the infrared branch under degraded visible-light conditions. Specifically, SpatialAdapt(·) is a learnable spatial adaptive operator that maps the illumination prior to a spatially varying gating map:

(8) $$\mathrm{SpatialAdapt}\left(I_{illum}\right)=\sigma \left({\mathrm{Conv}}_{3\times 3}^{\left(2\right)}\left(\phi \left({\mathrm{Conv}}_{3\times 3}^{\left(1\right)}\left(I_{illum}\right)\right)\right)\right),$$

where ϕ denotes the LeakyReLU activation.

It should be emphasized that IGAG is a learned soft-gating mechanism rather than a deterministic threshold-based rule. The formulation in Equation (7) does not impose a strict monotonic constraint for every individual image. Instead, the low-illumination prior encourages the network to increase the infrared contribution when the visible-light modality becomes less reliable, while the learnable MLP and SpatialAdapt branch allow the gate to remain spatially adaptive to the local scene content.

Therefore, the relationship between illumination and $w_{IR}$ should be interpreted as a statistical tendency guided by the module design, rather than as a hard mathematical guarantee. To empirically verify this behavior, we record $w_{IR}$ during inference and conduct an illumination-binned gate analysis in Section 4.6. The results show that the darker RGB groups received higher average infrared gate weights, supporting the intended illumination-adaptive behavior of IGAG.

This soft modulation facilitates dynamic compensation for visible-light degradation and enhances the stability of the fused representation under complex lighting conditions.

### 3.4. Frequency-Aware Fusion

Directly superimposing heterogeneous RGB–IR features after alignment and gating may still introduce undesirable responses. In particular, infrared features provide useful target contours under degraded visible-light conditions, but they may also contain stochastic thermal noise, false thermal responses, or background high-frequency fluctuations. Therefore, after IGAG estimates the illumination-dependent modality reliability, a further regulation step is required to prevent unreliable infrared details from being indiscriminately injected into visible-light textures.

To mitigate this issue, the Frequency-Aware Fusion (FAF) module adopts an approximate spatial-frequency decomposition strategy. Rather than employing strict frequency-domain transformations, such as Fourier or wavelet transforms, FAF uses lightweight spatial operations to obtain approximate base and detail components. This design is intended for efficient progressive fusion inside the MIRNet-v2 backbone, where a heavy frequency transform at each level would increase the computational cost and complicate end-to-end optimization. Specifically, FAF decouples multimodal feature streams into approximate low-frequency ($F^{low}$) and high-frequency ($F^{high}$) components and applies differentiated fusion strategies to each part.

**Figure 4:**
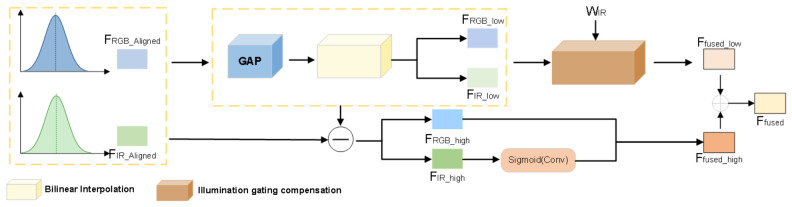
Detailed architecture of the Frequency-Aware Fusion (FAF) module. FAF performs lightweight spatial sub-band approximation by separating aligned features into base and detail components. The low-frequency branch uses the IGAG-generated gate $w_{IR}$ for illumination-aware compensation, while the high-frequency branch selectively injects infrared detail responses through a learned attention map, reducing indiscriminate thermal noise propagation.

As illustrated in Figure 4, FAF extracts $F^{low}$ through global average pooling (GAP) followed by bilinear interpolation to capture approximate base components, while obtaining $F^{high}$ via residual calculation to isolate detail variations. The low-frequency branch mainly models illumination and coarse structural consistency, whereas the high-frequency branch preserves local contours and texture responses. The fusion logic is formulated as follows:

(9) $$F_{fused}=F_{fused}^{low}+F_{fused}^{high},$$

(10) $$F_{fused}^{low}=w_{IR}\cdot F_{IR}^{low}+\left(1-w_{IR}\right)\cdot F_{RGB}^{low},$$

(11) $$F_{fused}^{high}=F_{RGB}^{high}+\sigma \left(\mathrm{Conv}\left(\left[F_{RGB}^{high};F_{IR}^{high}\right]\right)\right)⊙F_{IR}^{high}.$$

The low-frequency path employs illumination-gated weighted averaging to compensate for visible-light degradation and maintain smooth luminance transitions. In contrast, the high-frequency path adopts an attention-guided injection strategy; the infrared detail component is not directly added to the visible feature but is first modulated by a learned attention map generated from concatenated RGB–IR high-frequency features. This design allows the network to selectively introduce infrared contour information while suppressing unstable background noise and false high-frequency responses.

In this way, FAF serves as a regulation stage between illumination-guided modality weighting and the subsequent target-aware reinforcement module. It stabilizes the contour information extracted after the DAL and IGAG but does not globally amplify all infrared details. As shown in the ablation and qualitative results, this frequency-aware regulation helps reduce background disturbance and provides a cleaner structural basis for the final SGFD-R refinement.

### 3.5. Saliency-Guided Frequency Decoupling Reinforcement

Although the DAL, IGAG, and FAF provide aligned, illumination-adaptive, and frequency-regulated fused features, fine target boundaries may still be attenuated during deep feature propagation, especially for small, distant, or partially occluded road participants. This motivates SGFD-R as a final target-aware refinement stage. To mitigate the gradual weakening of boundary sharpness and the spectral imbalance caused by direct feature injection, the SGFD-R module shifts the fusion logic from global feature superposition toward target-aware local structure reinforcement.

In this work, the target-aware guidance in SGFD-R refers to a target-region prior inferred from infrared saliency, rather than semantic segmentation labels, object categories, or detector supervision. Since infrared saliency may also respond to non-target heat sources in roadside scenes, this prior is used as a soft spatial constraint rather than a hard semantic mask. This design is consistent with the detector-agnostic setting of ADS-MIR, where target-related structures are reinforced without coupling the enhancement module to a specific downstream detector.

**Figure 5:**
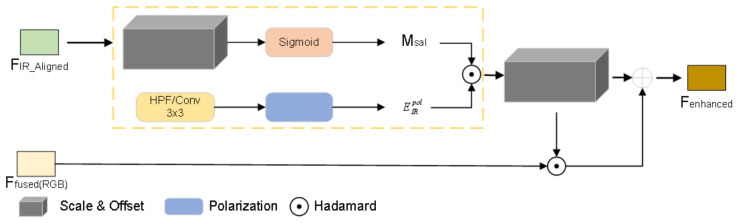
Detailed architecture of the Saliency-Guided Frequency Decoupling Reinforcement (SGFD-R) module. SGFD-R uses an infrared saliency-derived soft mask $M_{sal}$ to spatially constrain high-frequency reinforcement. The multiplicative path enhances local contrast, while the additive path injects localized residual structures, allowing target-related boundaries to be strengthened without globally amplifying all infrared responses.

As illustrated in Figure 5, the module first normalizes the input features. Based on the normalized intensity distribution of the infrared features (${\bar{F}}_{IR}$), it generates a continuous saliency mask $M_{sal}$ to highlight potential target regions:

(12) $$M_{sal}=\sigma \left(10.0\cdot \left({\bar{F}}_{IR}-0.5\right)\right).$$

Although the scaling factor makes the response relatively selective, $M_{sal}$ remains differentiable and does not perform binary foreground or background classification. Using this mask as a spatial constraint, a dual-path reinforcement model is applied to reconstruct the visible-light features:

(13) $$F_{enhanced}=\left(F_{RGB}⊙\left(1+\alpha \cdot {\bar{E}}_{IR}^{pol}⊙M_{sal}\right)\right)+\left(\beta \cdot {\bar{E}}_{IR}^{pol}⊙M_{sal}\right),$$

where ${\bar{E}}_{IR}^{pol}$ denotes the normalized polarized high-frequency component of the infrared modality, while α and β are learnable scaling parameters.

The multiplicative path controlled by α adaptively stretches local contrast around salient structures, while the additive path controlled by β injects localized high-frequency residuals to recover weakened target boundaries. Since both paths are modulated by $M_{sal}$, SGFD-R avoids globally amplifying all infrared high-frequency responses and reduces the risk of introducing excessive background artifacts.

This saliency-constrained residual formulation helps preserve the discriminative edge gradients required for high-level perception. Nevertheless, because infrared saliency may also respond to non-target heat sources, SGFD-R should be regarded as a soft saliency-guided reinforcement mechanism rather than a perfect thermal artifact filtering module. This limitation is further discussed in the Section 5.

### 3.6. Multi-Level Progressive Fusion Strategy

Conventional “single-pass” fusion paradigms typically execute cross-modal interaction only at the terminal stage of the backbone network. This often results in the loss of shallow spatial details and leaves deep feature contamination unresolved.In visible-infrared roadside perception, modality discrepancy, illumination-dependent reliability, and target-boundary degradation may appear at different feature depths. Therefore, relying only on terminal fusion is insufficient to simultaneously handle spatial consistency, frequency regulation, and structural reinforcement. To overcome these limitations, the ADS-MIR framework transforms the fusion logic from a single-point settlement into a hierarchical progressive integratio.

As illustrated in Figure 1, ADS-MIR embeds a progressive fusion chain after each of the four recursive residual groups (RRGs). The interaction logic at each level l∈{1,2,3,4} follows a cascaded path from alignment to structural reinforcement:

(14) $$F_{inter}^{\left(l\right)}={\mathrm{FAF}}^{\left(l\right)}\left({\mathrm{DAL}}^{\left(l\right)}\left(F_{RGB}^{\left(l\right)},F_{IR}^{\left(l\right)}\right),w_{IR}\right),$$

(15) $$F_{fused}^{\left(l\right)}={\mathrm{SGFD}-R}^{\left(l\right)}\left(F_{inter}^{\left(l\right)}\right),$$

(16) $$F_{RGB}^{\left(l+1\right)}=\sigma \left(F_{RGB}^{\left(l\right)}⊕F_{fused}^{\left(l\right)}\right),$$

where *l* denotes the RRG level index, $w_{IR}$ denotes the illumination-guided infrared gate generated by IGAG, and ⊕ represents feature aggregation. This formulation summarizes the functional progressive fusion chain across the backbone hierarchy; aligned RGB–IR representations are first regulated by FAF under the guidance of $w_{IR}$, then refined by SGFD-R, and finally injected into the succeeding RRG level as a calibration signal.

In this hierarchical structure, the intermediate feature $F_{inter}^{\left(l\right)}$ is synthesized through spatial-frequency approximation and subsequently refined by the SGFD-R module to reinforce target-related local structures. The resulting composite feature $F_{fused}^{\left(l\right)}$ serves as a prior calibration signal that is injected into the input of the succeeding RRG layer.

The roles of different levels are complementary. Shallow units mainly contribute to spatial consistency and contour stabilization, while deeper units accumulate illumination-aware and saliency-guided structural cues for target-boundary reinforcement. This progressive design mitigates the risk that useful infrared structural cues are weakened by repeated convolutional propagation before reaching the final reconstruction layer.

Through this multi-level progressive self-calibration, ADS-MIR gradually refines multimodal representations across the network hierarchy. This strategy helps maintain logical connectivity and structural integrity as features evolve throughout the network, rather than relying on a single terminal fusion operation to recover all lost details.

## 4. Experiments

### 4.1. Implementation Details

To ensure reproducibility, the proposed ADS-MIR framework was implemented in Python 3.9 using PyTorch 2.1.0 and torchvision 0.16.0. All experiments were conducted on Ubuntu 20.04 LTS. The training phase was executed on a server equipped with an Intel^®^ Core™ i9-14900KF CPU, 128 GB of DDR4 memory, and an NVIDIA RTX A6000 GPU with 48 GB of VRAM.

All paired RGB–IR inputs were resized or cropped to a fixed spatial resolution of 256×256 during training and inference. Unless otherwise specified, ADS-MIR was trained under the global image restoration objective described in Section 3 without using detector-specific loss functions. This setting is consistent with the detector-agnostic design of ADS-MIR, where the enhanced outputs can be used as preprocessing inputs for different downstream perception models.

To reduce early-stage feature degradation, we adopted a staged training strategy. The RGB restoration branch was first stabilized by loading a low-light, single-modal pretrained model or weights inherited from the previous training stage, and newly introduced components were then progressively inserted on top of the stabilized backbone. For initialization, the RGB branch used pretrained or inherited weights when available, and the IR branch used Kaiming initialization with a small random perturbation when trained from scratch. In the DAL, InstanceNorm2d (affine = True) followed the default affine initialization, i.e., γ=1 and β=0. Convolutional layers in IGAG, FAF, and SGFD-R were initialized using Kaiming initialization with zero bias. These settings helped stabilize early optimization and reduce premature high-frequency over-amplification.

The complete ADS-MIR architecture consists of 12.79 M trainable parameters. Under the fixed 256×256 RGB–IR input setting, ADS-MIR requires 296.87 G MACs, corresponding to 593.75 G FLOPs when one MAC is counted as two floating-point operations. The parameter count was computed by summing all trainable parameters, while MACs were profiled using the complete ADS-MIR forward path. Since FLOPs are sensitive to the input resolution and counting convention, the reported operations should be interpreted under this specific profiling setting. To evaluate practical deployment potential, inference speed benchmarking was conducted on an NVIDIA GeForce RTX 5090 GPU using the same 256×256 input resolution. ADS-MIR achieved an average inference time of approximately 44.56 ms per frame (standard deviation: 0.14 ms), corresponding to 22.4 frames per second (FPS). CUDA 12.1 and cuDNN 8.9.2 were used for acceleration.

### 4.2. Dataset

To validate the effectiveness of the ADS-MIR framework under different complex conditions, this study used two representative multimodal benchmark datasets with diverse target classes:

1. LLVIP dataset [26]: This dataset contains 15,488 pairs of strictly synchronized visible-light and infrared images, each with a resolution of 1920×1080. It primarily covers nighttime intelligent transportation sensing scenarios, where visible-light images suffer from severely degraded sensor performance due to extremely low light levels. This provides an appropriate environment for evaluating the model’s ability to reconstruct physical features under extremely low signal-to-noise ratios.
2. M3FD dataset [27]: This dataset covers a variety of challenging weather and illumination conditions, including sunny, rainy, foggy, and high-glare environments, and comprises a total of 4200 image pairs. Beyond multimodal images, it provides exhaustive bounding box annotations for six traffic-related object categories (i.e., people (11,478), cars (18,296), buses (700), trucks (1008), motorcycles (521), and lamps (2405)). This dense distribution of common road participants provides a solid foundation for evaluating the impact of enhanced results on subsequent perception tasks.

### 4.3. Evaluation Metrics

To objectively evaluate the reconstruction quality of the ADS-MIR framework, this study selected evaluation metrics across three dimensions: information abundance, edge texture, and perceptual fidelity. Specifically, these included the entropy (EN), average gradient (AG), spatial frequency (SF), standard deviation (SD), visual information fidelity (VIF), and edge information transfer factor ($Q^{AB/F}$). The detailed descriptions and mathematical definitions are as follows.

Information entropy (EN) was used to quantify the amount of information contained in the enhanced image $\hat{I}$, reflecting the overall information volume of the scene. Its calculation is defined as follows:

(17) $$EN=-\sum_{i=0}^{L-1}p_{i}\log_{2}p_{i}$$

where *L* denotes the total number of gray levels (typically 256) and $p_{i}$ represents the probability distribution of gray level *i* appearing in the enhanced image.

The average gradient (AG) measures the ability of an image to express fine textures, reflecting the variations in structural details and local clarity. It is calculated as follows:

(18) $$AG=\frac{1}{\left(M-1)(N-1\right)}\sum_{x=1}^{M-1}\sum_{y=1}^{N-1}\sqrt{\frac{\Delta I_{x}^{2}+\Delta I_{y}^{2}}{2}}$$

where $\Delta I_{x}$ and $\Delta I_{y}$ represent the finite differences of the pixel at (x,y) in the horizontal and vertical directions, respectively, and M×N denotes the image resolution.

The spatial frequency (SF) quantifies the overall activity of an image in the spatial domain, serving to evaluate the overall spatial activity and detail variation. The SF is determined by the combination of the row frequency (RF) and column frequency (CF) and is calculated as follows:

(19) $$RF=\sqrt{\frac{1}{MN}\sum_{x=1}^{M}\sum_{y=2}^{N}{\left[\hat{I}\left(x,y\right)-\hat{I}\left(x,y-1\right)\right]}^{2}}$$

(20) $$CF=\sqrt{\frac{1}{MN}\sum_{x=2}^{M}\sum_{y=1}^{N}{\left[\hat{I}\left(x,y\right)-\hat{I}\left(x-1,y\right)\right]}^{2}}$$

(21) $$SF=\sqrt{RF^{2}+CF^{2}}$$

where $\hat{I}\left(x,y\right)$ represents the pixel intensity of the enhanced image at coordinates (x,y).

The standard deviation (SD) measures the overall contrast and intensity distribution of the fused image. It is mathematically defined as follows:

(22) $$SD=\sqrt{\frac{1}{MN}\sum_{x=1}^{M}\sum_{y=1}^{N}{\left(\hat{I}\left(x,y\right)-\mu \right)}^{2}}$$

where μ denotes the mean intensity value of the entire image. A higher SD value indicates a wider distribution of gray levels, which typically corresponds to higher visual contrast and a broader dynamic range.

The visual information fidelity (VIF) reflects how well an image is suited for human vision by quantifying the mutual information between the enhanced image and the original scene. Its mathematical definition is

(23) $$VIF=\frac{\sum_{j\in subbands}I\left({\vec{C}}^{j};{\vec{F}}^{j}|s^{j}\right)}{\sum_{j\in subbands}I\left({\vec{C}}^{j};{\vec{E}}^{j}|s^{j}\right)}$$

where $I({\vec{C}}^{j};{\vec{E}}^{j}|s^{j})$ represents the mutual information extracted by the human visual system from the original reference signal and $I({\vec{C}}^{j};{\vec{F}}^{j}|s^{j})$ represents the information extracted from the enhanced image.

The edge information transfer factor ($Q^{AB/F}$) is an important indicator for evaluating structural preservation, as it is used to quantify the amount of edge information extracted from the source features $F_{RGB}$ and $F_{IR}$ and transferred to the enhanced image. Its value ranges from 0 to 1. It is calculated as follows:

(24) $$Q^{AB/F}=\frac{\sum_{x=1}^{M}\sum_{y=1}^{N}\left(Q_{x,y}^{AF}w_{x,y}^{A}+Q_{x,y}^{BF}w_{x,y}^{B}\right)}{\sum_{x=1}^{M}\sum_{y=1}^{N}\left(w_{x,y}^{A}+w_{x,y}^{B}\right)}$$

where $Q^{AF}$ and $Q^{BF}$ denote the edge preservation factors relative to the visible-light feature $F_{RGB}$ and infrared feature $F_{IR}$, respectively, and $w^{A}$ and $w^{B}$ represent the pixel-level saliency weights. This metric reflects the model’s ability to retain target contours and gradient boundaries.

It should be noted that these metrics emphasize different aspects of image quality. The EN, SD, AG, and SF mainly reflect information abundance, contrast distribution, and spatial activity, whereas the VIF is more closely related to human visual fidelity and perceptual naturalness. In contrast, $Q^{AB/F}$ focuses on the transfer of edge and gradient information from the visible and infrared source modalities to the enhanced image. Since ADS-MIR is designed for machine perception-oriented enhancement, improvements in structure-oriented metrics such as $Q^{AB/F}$ may not always coincide with higher VIF, especially in complex glare or foggy scenes, where target-boundary reinforcement can reduce perceptual smoothness. Therefore, VIF and $Q^{AB/F}$ are interpreted jointly in this work to analyze the trade-off between human visual naturalness and machine-oriented structural discriminability.

### 4.4. Comparative Experiments

To demonstrate the capabilities of ADS-MIR under complex intelligent transportation sensing conditions, we conducted quantitative comparative experiments on the LLVIP and M3FD datasets. Several representative state-of-the-art methods were selected as benchmarks, including DenseFuse [28], DDcGAN [29], GANMcC [30], RFN-Nest [31], SDNet [32], and MFEIF [33]. All methods were evaluated under a unified experimental setting to ensure a fair comparison.

**Figure 6:**
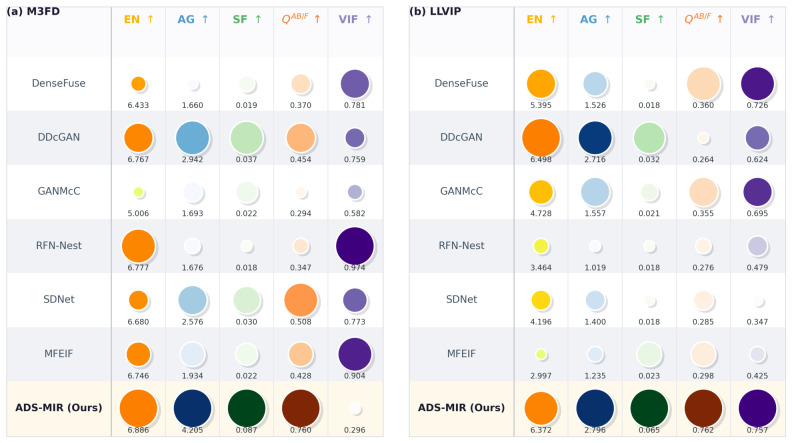
Quantitative comparison of image enhancement models. (**a**) Metric results on the M3FD dataset. (**b**) Metric results on the LLVIP dataset. Higher values generally indicate stronger performance for the reported metrics, while VIF and $Q^{AB/F}$ are interpreted according to their different emphases on perceptual fidelity and structural edge transfer. The upward arrows indicate that higher metric values correspond to better performance.

Figure 6 presents a multi-dimensional quantitative comparison across both benchmark datasets. As shown in Figure 6a, ADS-MIR achieved strong performance on the M3FD dataset in structure-related metrics such as the spatial frequency (SF) and edge information transfer factor ($Q^{AB/F}$). Specifically, ADS-MIR obtained a $Q^{AB/F}$ score of 0.760, corresponding to an improvement of approximately 49.6% over the strongest baseline of SDNet (0.508). This indicates that ADS-MIR is particularly effective in preserving target-related edge structures under complex roadside conditions involving fog, glare, and multimodal sensor heterogeneity.

Discussion on Optimization Preferences (VIF vs. $Q^{AB/F}$): While ADS-MIR achieved strong edge transfer performance on M3FD, its VIF score (0.296) was lower than those for smoothing-oriented methods such as RFN-Nest (0.974). This divergence reflects the different emphases of the two metrics; VIF favors human visual fidelity and perceptual naturalness, whereas $Q^{AB/F}$ measures edge and gradient transfer from source modalities. Since ADS-MIR is designed for machine perception-oriented enhancement, it prioritizes target contours and high-frequency structural cues, which may reduce perceptual smoothness in glare or foggy scenes. Therefore, ADS-MIR is interpreted as favoring structural discriminability rather than purely smoothing-oriented visual naturalness.

The results on the LLVIP dataset, shown in Figure 6b, further demonstrate the structural reconstruction capability of ADS-MIR under extreme nighttime conditions. ADS-MIR led in terms of average gradient (AG), SF, $Q^{AB/F}$, and VIF. Unlike M3FD, LLVIP mainly contains synchronized low-light nighttime scenes with fewer complex glare and weather disturbances. In this setting, structural enhancement is more consistent with perceptual fidelity, which explains the concurrent top VIF score (0.757). Specifically, ADS-MIR achieved a $Q^{AB/F}$ value of 0.762, corresponding to a 111.67% relative improvement over DenseFuse (0.360), while the SF improved by 103.1% compared with DDcGAN.

To assess statistical stability, we computed 95% confidence intervals for ADS-MIR based on the image-level $Q^{AB/F}$ scores, where *N* denotes the number of test image pairs. ADS-MIR achieved $Q^{AB/F}=0.7596\pm 0.0026$ on M3FD (N=840) and $Q^{AB/F}=0.7638\pm 0.0040$ on LLVIP (N=3463), where the intervals denoted 95% confidence intervals rather than standard deviations. These results support the stability of ADS-MIR’s structural preservation performance across the test samples.

### 4.5. Ablation Study

**Figure 7:**
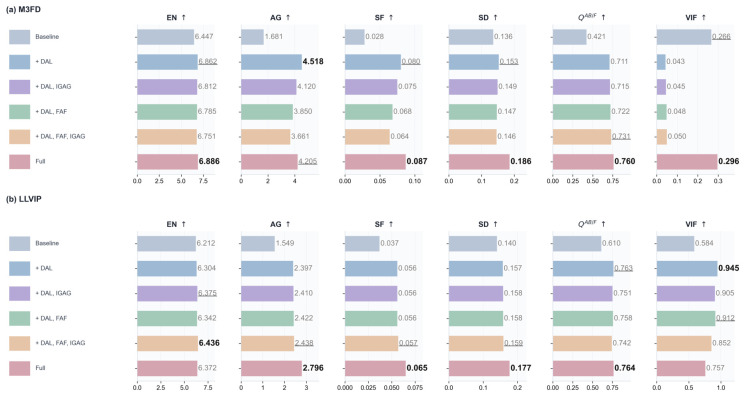
Ablation study results by configuration. (**a**) Metrics for M3FD. (**b**) Metrics for LLVIP. Within each metric column, the deepest color represents the best result, and the underlined value denotes the second-best performance.The upward arrows indicate that higher metric values correspond to better.

To evaluate the contribution of the domain alignment layer (DAL), Frequency-Aware Fusion (FAF), Illumination-Guided Adaptive Gating (IGAG), multi-level progressive fusion (MPF), and Saliency-Guided Frequency Decoupling Reinforcement (SGFD-R) module, this section uses a baseline dual-stream fusion network as Stage 0. Stage 1 introduces the DAL to examine feature-level statistical alignment; Stage 2 further integrates IGAG and FAF to evaluate illumination-adaptive gating and frequency-aware regulation; and Stage 3 represents the complete ADS-MIR model with all proposed components. This progressive setting followed the problem-driven design in Section 3; Stage 1 addresses cross-modal feature discrepancy, Stage 2 regulates illumination-dependent modality contribution and frequency responses, and Stage 3 reinforces target-related high-frequency boundaries through saliency-guided structural refinement. Figure 7 presents the quantitative results on M3FD and LLVIP.

As shown in Figure 7a, on the M3FD dataset, introducing the DAL significantly improved $Q^{AB/F}$ from 0.421 to 0.711, confirming the necessity of feature-level statistical alignment before cross-modal fusion. The DAL-only variant also exhibited an exceptionally high average gradient (AG: 4.518), which may reflect both useful high-frequency enhancement and undesirable infrared thermal noise. After IGAG and FAF were integrated, the fusion process shifted toward illumination-guided and frequency-decoupled regulation, helping suppress background disturbance while preserving useful contour information. The complete ADS-MIR model achieved the best performance among the evaluated configurations in terms of spatial frequency (SF: 0.087), standard deviation (SD: 0.186), and edge transfer ($Q^{AB/F}:0.760$), indicating that MPF and SGFD-R further strengthened target-related structural preservation.

On the LLVIP dataset, shown in Figure 7b, the final Stage 3 model pushed $Q^{AB/F}$ to 0.764. The individual gains of IGAG and FAF were relatively modest because most LLVIP samples already strongly favor the infrared modality, but their synergy with MPF and SGFD-R improved structural reinforcement. Although the entropy (EN) and VIF slightly decreased compared with Stage 2, this reflects a trade-off between visual naturalness and machine-oriented structural preservation rather than a simple degradation of enhancement quality. The complete model prioritizes structural gradients and edge transfer, thereby providing stronger target-boundary cues for downstream machine perception.

### 4.6. Inference-Time Validation of IGAG

To further verify whether the Illumination-Guided Adaptive Gating (IGAG) module learns the intended illumination-adaptive behavior, we conducted an inference-time gate analysis on the M3FD test set. During inference, we recorded the infrared gate weight $w_{IR}$ generated by IGAG and computed its average response for each image. Meanwhile, the mean RGB intensity of each visible-light input was used as an illumination indicator.

Specifically, all M3FD test samples were divided into four illumination groups according to the quartiles of their mean RGB intensity: bright, normal, dim, and extreme dark. For each group, we calculated the average RGB intensity and the mean infrared gate weight. The purpose of this analysis was to examine whether darker visible-light conditions statistically corresponded to larger infrared branch weights.

**Figure 8:**
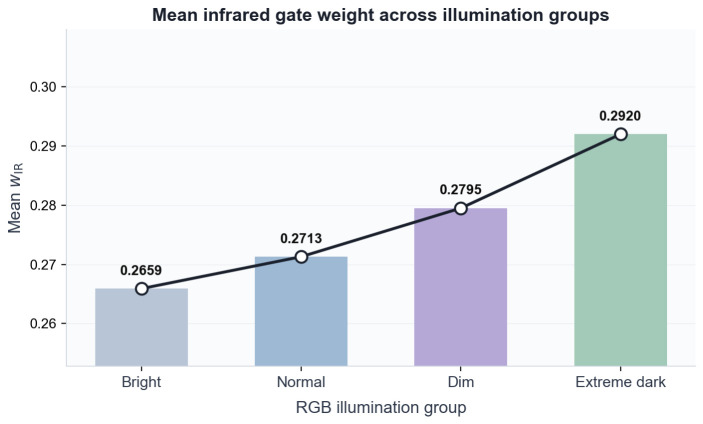
Inference-time validation of IGAG on the M3FD test set. Samples were divided into four illumination groups according to quartiles of mean RGB intensity. The average infrared gate weight $w_{IR}$ increased from the bright group to the extreme dark group, indicating that IGAG statistically tends to assign larger infrared weights under less visible-light illumination.

As shown in Figure 8, the average infrared gate weight increased as RGB illumination decreased. Specifically, the mean $w_{IR}$ rose from 0.2659 in the bright group to 0.2713 in the normal group, 0.2795 in the dim group, and 0.2920 in the extreme dark group. This result empirically supports the intended illumination-adaptive behavior of IGAG, showing that the network tends to rely more on infrared cues when visible-light observations become less reliable.

It should be emphasized that IGAG is a learned soft-gating mechanism rather than a deterministic threshold-based rule. Therefore, the observed relationship between illumination and $w_{IR}$ should be interpreted as a statistical tendency over the test set, rather than a strict monotonic guarantee for every individual image. This analysis provides empirical evidence for the low-illumination prior introduced in Equation (7).

### 4.7. Downstream Object Detection Validation

To examine how the enhanced inputs influence downstream perception, we evaluated object detection performance using GM-DETR [34] as the reference detector. We compared the detection accuracy between raw low-light inputs and ADS-MIR-enhanced inputs.

On the M3FD dataset, which contains complex conditions such as glare and dense fog, GM-DETR achieved an ${\mathrm{mAP}}_{50}$ of 0.857 and an mAP of 0.568 on raw bimodal inputs. With ADS-MIR-enhanced inputs, these metrics increased to 0.867 and 0.570, respectively, indicating that ADS-MIR provides moderate downstream gains by improving structural discriminability in challenging scenes.

**Figure 9:**
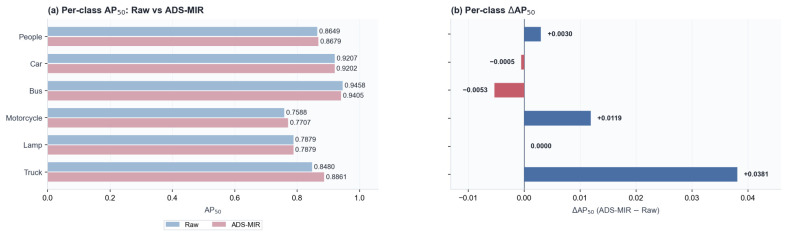
Class-wise detection analysis on M3FD. (**a**) AP50 comparison between raw inputs and ADS-MIR-enhanced inputs. (**b**) Class-wise AP50 changes after ADS-MIR enhancement, where positive values indicate improved detection accuracy compared with raw inputs. ADS-MIR brought the clearest gains for Truck and Motorcycle, while categories with already-high baseline accuracy showed limited headroom or minor fluctuations.

To identify which object categories benefited most, we visualized the class-wise AP50 results on M3FD in Figure 9. Figure 9a compares AP50 before and after enhancement, while Figure 9b shows the corresponding AP50 changes. The improvement was category-dependent; ADS-MIR brought the clearest gain for trucks (+0.0381 AP50), followed by motorcycles (+0.0119 AP50) and people (+0.0030 AP50). Cars and lamps remained nearly unchanged, while buses showed a slight decrease. These results suggest that ADS-MIR mainly benefits categories with reliable structural or thermal contours rather than uniformly improving every class.

On the LLVIP dataset, which represents extremely dark nighttime scenarios, ADS-MIR also improved GM-DETR performance. The baseline detection results (${\mathrm{mAP}}_{50}$: 0.974, mAP: 0.698) increased to an ${\mathrm{mAP}}_{50}$ of 0.979 and an mAP of 0.702 after enhancement. Overall, ADS-MIR provided moderate but consistent downstream detection gains, especially for hard samples and categories with reliable structural or thermal cues. This is consistent with its detector-agnostic design, which improves input structural quality without directly optimizing detector-specific losses.

### 4.8. Qualitative Evaluation and Visualization

**Figure 10:**
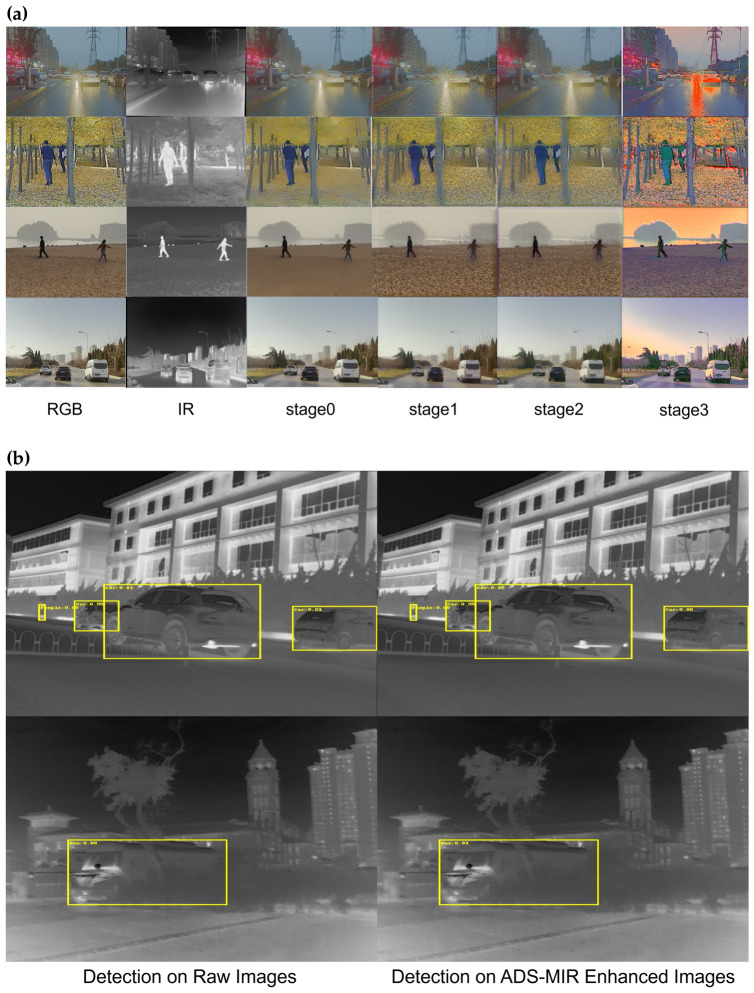
Qualitative visualization analysis. (**a**) Evolution from Stage 0 to Stage 3, demonstrating progressive artifact suppression, contour stabilization, and target-boundary reinforcement. (**b**) Detection performance on raw low-light images versus ADS-MIR-enhanced images, illustrating performance gains on certain hard samples in edge cases.

To visually demonstrate the reconstruction behavior of ADS-MIR, this section presents the qualitative results for both the internal enhancement stages and downstream object detection, as shown in Figure 10. Figure 10a illustrates the progressive enhancement process on representative M3FD samples. The Stage 0 baseline increased the brightness but also introduced noticeable background noise. After the DAL was introduced in Stage 1, ghosting artifacts were reduced, and object contours became more stable. Stage 2, with FAF and IGAG, produced smoother texture transitions and suppressed part of the glare-related disturbance. In Stage 3, SGFD-R further reinforced target-related edge details, consistent with the structural metric improvements reported in Section 4.5.

This qualitative evolution is consistent with the problem-driven design of ADS-MIR; the DAL stabilizes cross-modal alignment, IGAG and FAF regulate illumination-dependent responses, and SGFD-R reinforces target-related boundaries. These observations complement the quantitative ablation results by showing how each stage contributes to artifact reduction and structural preservation.

Figure 10b compares the GM-DETR [34] detection results on raw and ADS-MIR-enhanced inputs. ADS-MIR improved target visibility in certain hard samples, where heavily obscured targets that were missed or assigned near-zero confidence in raw images were detected with higher confidence after enhancement. These examples are interpreted as complementary evidence rather than a replacement for quantitative detection metrics, since the overall detection benefit remained moderate, category-dependent, and detector-agnostic, as discussed in Section 4.7.

## 5. Discussion and Conclusions

### 5.1. Summary of Core Insights

This study addresses the reliability challenges of single-modal vision systems under complex lighting conditions in intelligent transportation systems. We proposed ADS-MIR, a machine perception-oriented multimodal image enhancement framework. Rather than solely prioritizing human visual aesthetics, the framework focuses on cross-modal alignment, illumination-adaptive feature modulation, frequency-aware fusion, and saliency-guided structural reinforcement to preserve critical edge gradients for downstream algorithms.

Quantitative evaluations revealed that ADS-MIR achieved a relative improvement from 49.6% to 111.67% in terms of the edge information transfer factor ($Q^{AB/F}$) across the M3FD and LLVIP datasets, indicating a distinct advantage in structural preservation and target contour restoration. The image-level confidence interval analysis further shows that the $Q^{AB/F}$ performance of ADS-MIR remained statistically stable on both datasets, supporting the reliability of the observed structural preservation gains. Furthermore, downstream object detection validations indicated that the enhanced representations provided more discriminative inputs, leading to moderate but consistent performance gains and showing positive effects on mitigating missed detections for difficult samples in complex scenes. The class-wise detection analysis further suggests that the benefit was category-dependent, with clearer gains for objects whose structural or thermal contours could be reliably reinforced. These findings suggest that strategically prioritizing structural fidelity over smoothing-oriented visual naturalness is a viable paradigm for improving machine perception in challenging intelligent transportation sensing environments.

### 5.2. Limitations

Although ADS-MIR demonstrated substantial efficacy in structural preservation, several limitations should be noted.

First, the dual-stream, multi-level progressive architecture introduces considerable computational complexity. Under the 256×256 RGB–IR input setting, ADS-MIR contains 12.79 M trainable parameters and requires 593.75 G FLOPs, achieving 22.4 FPS on an RTX 5090 GPU. Although this indicates near real-time potential on high-performance hardware, processing high-resolution multimodal streams concurrently remains challenging for resource-constrained roadside units (RSUs).

Second, ADS-MIR currently functions as an independent preprocessing module. While this detector-agnostic design improves modularity and allows ADS-MIR-enhanced outputs to be used with different perception backends, the absence of direct gradient backpropagation from downstream detectors means that the enhancement process is not explicitly optimized for the specific loss landscape of the target task. This may partially explain why the downstream detection gains are moderate and category-dependent rather than uniformly large across all object classes.

Third, the saliency-guided reinforcement mechanism relies on infrared saliency priors to guide local structural enhancement. Although SGFD-R uses a soft saliency mask rather than a hard semantic mask, infrared responses may still be affected by non-target heat sources in practical roadside scenes, such as heated asphalt, building facades, vehicle exhaust regions, or thermal reflections. These false thermal responses may introduce local reinforcement bias in challenging cases.

Fourth, the current validation was conducted on LLVIP and M3FD. Although these datasets cover representative low-light, nighttime, foggy, rainy, glare, and traffic-related scenarios, they cannot exhaust all possible sensing configurations. Different camera baselines, thermal sensor types, calibration settings, weather domains, and roadside layouts may introduce additional distribution shifts. Broader cross-domain validation remains necessary before large-scale deployment.

Finally, an inherent perceptual-structural trade-off persists between human visual naturalness and machine feature fidelity. The local structure reinforcement executed by SGFD-R improves edge information transfer ($Q^{AB/F}$) but may compromise visual information fidelity (VIF) in some complex scenes. In human-in-the-loop applications, such enhanced outputs may appear less visually natural despite providing stronger structural cues for machine perception.

### 5.3. Future Work Outlook

While ADS-MIR demonstrated robust performance in complex environments, further investigation is required to enhance its cross-scenario adaptability, deployment efficiency, and task-specific synergy. Future research will focus on the following directions:

- Lightweight Design and Dynamic Execution: We aim to explore lightweight architectures, pruning, quantization, and knowledge distillation to improve deployment efficiency on resource-constrained roadside units (RSUs). In addition, illumination-aware dynamic execution will be investigated to adaptively bypass computationally intensive paths under favorable lighting conditions.
- Task-Driven Joint Optimization: Future iterations will explore deeper coupling between ADS-MIR and downstream detectors. By introducing task-consistency constraints, detector feedback, or end-to-end fine-tuning mechanisms, the framework may better align low-level structural restoration with high-level perception decisions while preserving deployment flexibility.
- Robustness and Cross-Domain Generalization: Future work will investigate explicit thermal artifact suppression, temporal consistency modeling, and broader RGB–IR validation across different sensor baselines, calibration qualities, thermal camera types, and adverse weather domains. This will help reduce the influence of non-target heat sources and evaluate the robustness of ADS-MIR under more diverse deployment conditions.
- Balancing Structural Discriminability and Image Naturalness: We plan to investigate multi-objective optimization strategies to improve the chromatic naturalness of restored images without compromising the gains in structural preservation. This will allow the framework to better satisfy both automated machine analysis and human visual monitoring requirements.

## Data Availability

The datasets analyzed during this study are publicly available. The LLVIP dataset [26] is accessible via its official project page (https://bupt-ai-cz.github.io/LLVIP/, accessed on 28 May 2026). The M3FD dataset [27] can be obtained from the repository provided by the original authors (https://github.com/JinyuanLiu-CV/TarDAL, accessed on 28 May 2026). Both datasets were utilized in accordance with their respective open-access licenses. No new data were generated during this research.
